# Migraine and sleep disorders: a systematic review

**DOI:** 10.1186/s10194-020-01192-5

**Published:** 2020-10-27

**Authors:** Cindy Tiseo, Alessandro Vacca, Anton Felbush, Tamara Filimonova, Annalisa Gai, Tatyana Glazyrina, Irina Anna Hubalek, Yelena Marchenko, Lucas Hendrik Overeem, Serena Piroso, Alexander Tkachev, Paolo Martelletti, Simona Sacco, on behalf of the European Headache Federation School of Advanced Studies (EHF-SAS)

**Affiliations:** 1 Department of Applied Clinical Sciences and Biotechnology, University of L’Aquila, L’Aquila, Italy; 2 Regional Referral Headache Centre, S.S. Filippo e Nicola Hospital, Avezzano, L’Aquila, Italy; 3 Headache Center, Department of Neuroscience “Rita Levi Montalcini”, University of Torino, Torino, Italy; 4 Pain Treatment Center, OOO “Vertebra”, Samara City, Russia; 5 Federal State Budget Educational Institution of Higher Education “Academician Ye. A. Vagner Perm State Medical University” of the Ministry of Healthcare of the Russian Federation, Perm, Russia; 6 LLC Stomat Business Company, Moscow, Russia; 7 Department of Neurology, Headache Center, Charité University Medicine Berlin, Berlin, Germany; 8 V. A. Almazov National Medical Research Centre, Saint-Petersburg, Russia; 9 Charité – Universitätsmedizin Berlin Charité Centrum Neurologie, Neurochirurgie und Psychiatrie CC, Berlin, Germany; 10 Department of Human Neurosciences, Sapienza University of Rome, Roma, Italy; 11 Department of Neurology, Neurosurgery, medical genetics, I.M. Sechenov First Moscow State Medical University, Moscow, Russia; 12 Department of Clinical and Molecular Medicine, Sapienza University of Rome, Roma, Italy; 13 Regional Referral Headache Centre, Sant’Andrea Hospital, Rome, Italy

**Keywords:** Headache, Migraine, Sleep disorders, Insomnia, Narcolepsy, Restless leg syndrome, Sleep apnea, Periodic limb movement disorder, Circadian rhythm sleep-wake disorders, Parasomnias

## Abstract

Migraine and sleep disorders are common and often burdensome chronic conditions with a high prevalence in the general population, and with considerable socio-economic impact and costs.

The existence of a relationship between migraine and sleep disorders has been recognized from centuries by clinicians and epidemiological studies. Nevertheless, the exact nature of this association, the underlying mechanisms and interactions are complex and not completely understood. Recent biochemical and functional imaging studies identified central nervous system structures and neurotransmitters involved in the pathophysiology of migraine and also important for the regulation of normal sleep architecture, suggesting a possible causative role, in the pathogenesis of both disorders, of a dysregulation in these common nervous system pathways.

This systematic review summarizes the existing data on migraine and sleep disorders with the aim to evaluate the existence of a causal relationship and to assess the presence of influencing factors. The identification of specific sleep disorders associated with migraine should induce clinicians to systematically assess their presence in migraine patients and to adopt combined treatment strategies.

## Introduction

Migraine and sleep disorders are common and often burdensome chronic conditions with a high prevalence in the general population [1–3]. Those disorders often coexist, and this has led to hypothesize an association not only driven by chance occurrence. In fact, some studies support the evidence that migraineurs have worse sleep quality than non-migraineurs [4–16], that self-reported poor sleep quality is associated with increased frequency of attacks or chronification of migraine [17–20], and that preventive migraine treatments might improve the quality of sleep [4, 12].

Despite extensive investigations, the exact nature and direction of the association remains enigmatic; migraine may be the result of sleep disruption, but also sleep disruption may trigger migraine, or migraine and sleep disruption may be symptoms of an unrelated medical condition, or they might be two intrinsically related phenomena with shared pathophysiological mechanisms [21].

**Fig. 1:**
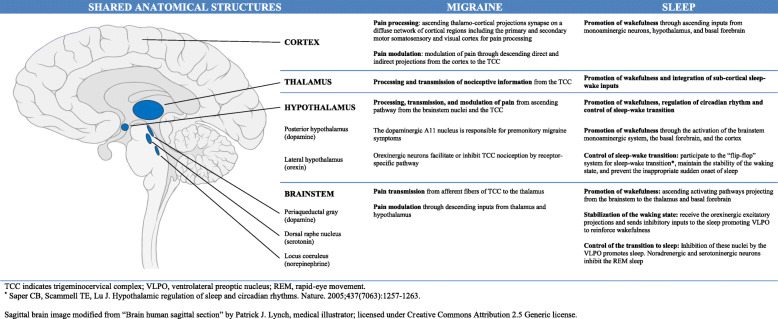
Key structures involved in migraine and sleep-wake regulation

Recent biochemical and functional imaging studies identified central nervous system structures and neurotransmitters involved in the pathophysiology of migraine and also important for the regulation of normal sleep architecture, suggesting a possible causative role, in the pathogenesis of both disorders, of a dysregulation in these common nervous system pathways [21, 22] (Fig. 1). Available evidence suggests that diencephalic and brainstem regions are the main anatomical structures involved in migraine pathogenesis and in sleep-wake cycle regulation, and orexins, melatonin, pituitary adenylate cyclase-activating polypeptide, serotonin, dopamine and adenosine are the most studied molecules for their possible role as mediators of this relationship [21, 22].

This review has the aim to elucidate the current evidence about the association between migraine and sleep disorders in order to improve the treatment strategies of the two conditions and the understanding of the common pathophysiology.

## Methods

This systematic review was performed according to the Preferred Reporting Items for Systematic Reviews and Meta-Analyses (PRISMA) guidelines [23].

### Data sources, search strategy, and data extraction

Studies were identified by searching papers indexed on PubMed and Scopus. Two investigators (A.R. and S.P.) conducted an independent search on both databases using the search terms “headache” OR “migraine” AND “sleep”. The search was carried out from January 1, 1990 to November 30, 2018, and was restricted to humans, and to articles published in English language. Duplicate publications were removed by checking manually. Titles and abstracts were screened to verify study eligibility. Full texts of selected studies were evaluated if appropriate. The reference lists and Google Scholar citations for all primary and review articles were also searched. Discrepancies between reviewers were resolved by discussion.

### Study selection

Clinical trials, observational studies and case series including subjects of both sexes, aged ≥18 years, and of all ethnicities were eligible to be included in the study. Studies had to diagnose migraine according to the available International Classification of Headache Disorders (ICHD) [24–27] and to report a clear description of the adopted criteria for the diagnosis of the considered sleep disorder.

Study papers lacking a clear description of diagnostic criteria for migraine and for the included sleep disorders, including subjects with headaches other than migraine, lacking a clear definition of study design and setting, case reports, letters to the editor, published erratum, abstracts, studies not performed on humans, studies not written in English and unpublished studies were excluded.

Evidence about the association between migraine and sleep disorders derived from studies included in this systematic review has been organized according to the major diagnostic sections of the ICSD-third edition [28, 29] (Table 1**)**.

**Table 1:** Sleep disorders according to the International Classification of Sleep Disorders – third edition (Adapted from American Academy of Sleep Medicine. International Classification of Sleep Disorders. 3rd ed. Darien, IL: American Academy of Sleep Medicine; 2014 and from Sateia MJ. International classification of sleep disorders-third edition: highlights and modifications. Chest. 2014;146:1387–94.)

ICSD-third edition major diagnostic sections	Definition	Disorder
Insomnia	Insomnia is defined as a persistent difficulty with sleep initiation, duration, consolidation, or quality that occurs despite adequate opportunity and circumstances for sleep, and results in some form of daytime impairment. Daytime symptoms typically include fatigue, decreased mood or irritability, general malaise, and cognitive impairment.	Chronic insomnia disorder
		Short-term insomnia disorder
		Other insomnia disorder
Sleep-related breathing disorders	Sleep-related breathing disorders are characterized by abnormalities of respiration during sleep. In some of these disorders, respiration is also abnormal during wakefulness. Specific pulmonary or neurological disorder should be diagnosed separately, in association with a diagnosis of sleep-related hypoventilation or sleep-related hypoxemia.	Obstructive sleep apnea disorders (OSA) Obstructive sleep apnea, adult Obstructive sleep apnea, pediatric
		Central sleep apnea syndromes Central sleep apnea with Cheyne-Stokes breathing Central apnea due to a medical disorder without Cheyne-Stokes breathing Central sleep apnea due to high altitude periodic breathing Central sleep apnea due to a medication or substance Primary central sleep apnea Primary central sleep apnea of infancy Primary central sleep apnea of prematurity Treatment-emergent central sleep apnea
		Sleep-related hypoventilation disorders Obesity hypoventilation syndrome Congenital central alveolar hypoventilation syndrome Late-onset central hypoventilation with hypothalamic dysfunction Idiopathic central alveolar hypoventilation Sleep-related hypoventilation due to a medication or substance Sleep-related hypoventilation due to a medical disorder
		Sleep-related hypoxemia disorder
Sleep-related movement disorders	Sleep-related movement disorders are characterized by relatively simple, usually stereotyped, movements that disturb sleep or its onset. Restless legs syndrome (RLS) is an exception in that patients typically engage in walking or nonstereotypic limb movement to reduce leg discomfort. Nocturnal sleep disturbance or complaints of daytime sleepiness or fatigue are a prerequisite for a diagnosis of a sleep-related movement disorder.	Restless legs syndrome (RLS)
		Periodic limb movement disorder
		Sleep-related leg cramps
		Sleep-related bruxism
		Sleep-related rhythmic movement disorder
		Benign sleep myoclonus of infancy
		Propriospinal myoclonus at sleep onset
		Sleep-related movement disorder due to a medical disorder
		Sleep-related movement disorder due to a medication or substance
		Sleep-related movement disorder, unspecified
Central disorders of hypersomnolence	Central disorders of hypersomnolence are characterized by excessive daytime sleepiness (hypersomnolence) that is not attributable to another sleep disorder, specifically those that result in disturbed sleep (eg, sleep-related breathing disorders) or abnormalities of circadian rhythm. The cardinal feature of all the central disorders of hypersomnolence is a subjective complaint of excessive daytime sleepiness, defined as daily episodes of an irrepressible need to sleep or daytime lapses into sleep.	Narcolepsy type 1
		Narcolepsy type 2
		Idiopathic hypersomnia
		Kleine-Levin syndrome
		Hypersomnia due to a medical disorder
		Hypersomnia due to a medication or substance
		Hypersomnia associated with a psychiatric disorder
		Insufficient sleep syndrome
Circadian rhythm sleep-wake disorders	Circadian rhythm sleep-wake disorders are characterized by chronic or recurrent pattern of sleep-wake rhythm disruption primarily due to alteration of the endogenous circadian timing system or misalignment between the endogenous circadian rhythm and the sleep-wake schedule desired or required by an individual’s physical environment or social/work schedules.	Delayed sleep-wake phase disorder
		Advanced sleep-wake phase disorder
		Irregular sleep-wake rhythm disorder
		Non-24-h sleep-wake rhythm disorder
		Shift work disorder
		Jet lag disorder
		Circadian sleep-wake disorder not otherwise specified
Parasomnias	Parasomnias are undesirable physical events or experiences that occur during entry into sleep, within sleep, or during arousal from sleep. Parasomnias encompass abnormal sleep-related complex movements, behaviours, emotions, perceptions, dreams, and autonomic nervous system activity that may occur during the phase non-rapid eye movement (NREM) or rapid eye movement (REM), or during transitions to and from sleep.Parasomnias are clinical disorders because of the resulting injuries, sleep disruption, adverse health effects, and untoward psychosocial effects. The clinical consequences of the parasomnias can affect the patient, the bed partner, or both.	NREM-related parasomnias: Disorders of arousal Confusional arousals SleepwalkingSleep terrors Sleep-related eating disorder
		REM-related parasomnias: REM sleep behaviour disorder Recurrent isolated sleep paralysis Nightmare disorder
		Other parasomnias: Exploding head syndrome Sleep-related hallucinations Sleep enuresis Parasomnia due to a medical disorder Parasomnia due to a medication or substance Parasomnia, unspecified
Other sleep disorders	Sleep disorders that cannot be classified elsewhere in the ICSD-third edition.	

*ICSD* indicates International Classification of Sleep Disorders, *NREM* non-rapid eye movement, *OSA* obstructive sleep apnea, *REM* rapid eye movement, *RLS* restless legs syndrome

The ICSD is a comprehensive classification system of sleep disorders designed as a diagnostic and coding tool that is widely used for both clinical and epidemiological purposes. The first edition of the ICSD was produced in 1990 [30]; it has been revised and updated in 1997 (ICSD-R) [31], in 2005 (ICSD-second edition) [32], and in 2014 (ICSD-third edition) [28, 29].

The recently released ICSD-third edition includes sleep disorders categorized in 7 major diagnostic sections: insomnia, sleep-related breathing disorders, sleep-related movement disorders, central disorders of hypersomnolence, circadian rhythm sleep-wake disorders, parasomnias and other sleep disorders (Table 1). Each disorder is presented in detail with specific diagnostic criteria. In addition, the ICSD-third edition includes two appendices listing: (A) sleep-related medical and neurological disorders, and (B) the International Classification of Diseases, Tenth Revision, Clinical Modification (ICD-10-CM) codes for substance-induced sleep disorders.

## Insomnia

Insomnia is a frequent and often neglected sleep disorder occurring in individuals of all ages and races. Prevalence estimates vary according to the study design and the adopted definition of insomnia; from one- to two-thirds of adults have insomnia symptoms and approximately 10% to 15% meet a chronic insomnia diagnosis [33–38].

The association between migraine and insomnia has been evaluated in several epidemiological studies [39–48]. A significant higher prevalence of insomnia and insomnia complaints has been documented in patients with migraine compared to those without headache [39, 43, 47], and a higher prevalence of migraine has been reported in subjects with insomnia compared to those without [43].

According to the results of the Nord-Trøndelag Health (HUNT-2 and HUNT-3) prospective population-based study, the association between migraine and insomnia may be bidirectional. Indeed, compared to headache-free subjects without insomnia, headache-free individuals with insomnia had a higher risk of developing migraine (relative risk [RR], 1.4) 11 years later [40]. Similarly, individuals with migraine had a 2-fold increased risk (OR, 1.7) of developing insomnia 11 years later compared to subjects without, and this risk was higher in those with at least 7 migraine days/month (OR, 2.1 vs 1.7), and in those with comorbid chronic musculoskeletal complaints (OR, 2.2) [41]. The presence of insomnia is associated with increased migraine pain intensity [43, 45], impact [43, 44], attack frequency [44, 45] and risk of chronification [40, 46]. The observed association between insomnia and migraine was found to be not solely attributable to anxiety and depression [39, 48].

Nevertheless, the association may be unspecific for migraine since the prevalence of insomnia complaints, although higher in subjects with headache than in those without, did not differ by headache subtype [39, 42]. Contrarywise, Kim et al., found a higher prevalence of insomnia in subjects with migraine (25.9%) compared to those with non-migraine headache (15.1%) [43]. The results from longitudinal cohort studies further support the hypothesis that insomnia may be generally associated with headache, since the risk of insomnia was found to be similar in individuals with both migraine (OR, 1.9) and non-migraine headaches (OR, 1.7) [41], and individuals with insomnia had the same risk of developing migraine or non-migraine headache (RR, 1.4 for any headache; RR, 1.4 for tension-type headache; RR, 1.4 for migraine; RR, 1.4 for nonclassified headache) [40].

A double-blind, placebo-controlled, parallel-group study [49] randomized patients with migraine and insomnia to receive eszopiclone 3 mg at bedtime or placebo with the aim to test the role of insomnia on migraine frequency and severity. The study [49] failed to answer the question as to whether insomnia is a risk factor for increased headache frequency and headache intensity in migraineurs, since active treatment did not lead to improvement in the total sleep time compared to placebo. Furthermore, no differences were found in headache frequency, intensity, and duration, while only a reduction in night-time awakenings as well as in daytime fatigue in favour of eszopiclone were reported.

Cognitive behavioral therapy including sleep hygiene, relaxation training, stimulus control therapy, sleep restriction therapy and cognitive therapy has been proved to be effective on both insomnia complaints and comorbid symptoms and is the recommended first-line treatment for chronic insomnia in adults [50]. Recent evidence from clinical trials suggests that cognitive behavioral therapy for insomnia is effective in improving migraine attack frequency [51, 52] and pain intensity [52]. A sequential Bayesian analysis providing a quantitative synthesis of the results of these trials showed that cognitive behavioral therapy for insomnia decreased headache frequency by 6.2 (95% CI, − 9.7 to − 2.7) days more in patients with chronic migraine than in control group, supporting the effective role of cognitive behavioral therapy as a non-invasive adjunctive treatment for chronic migraine [53].

The pathological mechanism underlying the association between migraine and insomnia is not yet fully understood. According to most of the available studies, migraine attack onset follows a circadian variation, with an early morning or late-night peak of migraine attack onset [54]. The observed circadian pattern of migraine attack onset may be related to a temporal relationship with rapid eye movement (REM) sleep stages. Interestingly, nocturnal arousal from sleep with migraine has been found to be more likely during REM sleep [55, 56], and an increased REM sleep and REM latency has been documented by an electroencephalographic study [57].

The circadian variability and the temporal correlation with REM sleep stages of migraine attacks emphasize respectively a hypothalamic involvement and a brainstem dysfunction in networks involved in sleep stages regulation. On the other hand, chronic exposure to insufficient sleep has been reported to decrease habituation to painful stimuli and to induce alterations in pain inhibitory systems, possibly favoring headache chronification [58].

Hypothalamic and brainstem dysfunctions have been hypothesized as common pathological mechanisms of migraine and insomnia. These structures are involved in both sleep-wake physiology and pain transmission and modulation (Fig. 1), and their dysfunctional activity might explain the observed bidirectional relationship between migraine and insomnia.

**Fig. 2:**
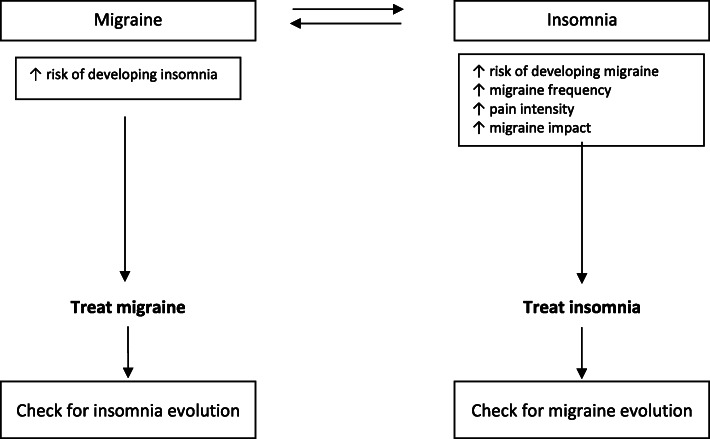
Interaction between migraine and insomnia. Available evidence suggests the existence of a bidirectional association between migraine and insomnia that is independent from anxiety and depression. Migraine patients are at increased risk of developing insomnia, and insomnia is a risk factor for migraine onset and for increased migraine impact, pain intensity, and chronification. Routine evaluation of the presence of insomnia complaints in patients with migraine and implementation of specific pharmacological and non-pharmacological insomnia treatments would be appropriate since a reduction of migraine burden might be observed

KEY MESSAGE: Overall, the available evidence suggests the existence of a bidirectional relationship between migraine and insomnia that is independent from anxiety and depression (Fig. 2). Insomnia is a risk factor for migraine onset and for increased migraine impact, pain intensity and chronification. Moreover, migraineurs are at increased risk of developing insomnia. Nevertheless, the association might not be specific for migraine since insomnia has been more generically associated with headache.

Clinicians should always look for insomnia complaints in patients with headache, especially in those with migraine, in order to manage this comorbid association through the implementation of specific insomnia treatment in the routine management of these patients; the choice might fall on those medications also approved for insomnia treatment, such as amitriptyline. Considering the potential effect of insomnia on the migraine burden, clinicians should also consider the implementation of non-pharmacological insomnia treatments, including behavioral sleep modification strategies.

## Sleep-related breathing disorders

Sleep-related breathing disorders are chronic pathological conditions characterized by repeated abnormal breathing during sleep, which lead to the fragmentation of sleep and decreases in oxyhemoglobin saturation. They are common sleep disorders with a prevalence estimated to be 20% in middle-aged adults [59, 60]. Men are from 2.0 to 3.7 times as likely as women to suffer from sleep-related breathing disorders [59].

OSA is characterized by repeated episodes of airflow reduction associated with oxygen desaturation and sleep disruption, caused by the partial (hypopnea) or complete (apnea) obstruction of the upper airways during sleep. These events often result in reductions of blood oxygen saturation and are usually terminated by brief arousals from sleep. Individuals with OSA more frequently experience awakening headaches (29%) and tension-type headache (15%), and less frequently migraine (8%) [61]. Population-based studies showed that the prevalence of OSA is similar among individuals with migraine and the general population without migraine [62], and among individuals with migraine without aura and migraine with aura [63]. Interestingly, a retrospective study on patients recruited from a neurology clinic for headaches, referred for a polysomnography (PSG) for presumed sleep-related breathing disorder, reported that chronic migraine without aura was predictive of OSA presence [64].

The treatment of OSA syndrome with continuous positive airway pressure has been associated with an improvement of sleep quality and migraine in terms of reduction of the mean frequency of attacks per month (from 5.8 to 0.1 days), mean attack duration, pain intensity, mean number of days with inability to work, and acute medication intake [65].

Obesity is considered a major risk factor for the development and progression of OSA [60, 66–68]. In the adult population, the prevalence of OSA is estimated to be about 25%, and as high as 45% in obese subjects. Furthermore, patients with mild OSA who gain 10% of their body weight are at a six-fold increased risk of progression of OSA, and an equivalent weight loss can result in a more than 20% improvement in OSA severity [68]. On the other hand, obese individuals are at increased risk for migraine [69] and in particular for chronic migraine [70]. Those interesting findings should stimulate further research aimed at exploring the role of obesity in the association between OSA and migraine and the effect of body weight management in patients with those two comorbid pathological conditions. Moreover, it would be interesting to evaluate if obesity represents a shared risk factor for migraine and OSA, or if migraine, obstructive sleep apnea and obesity represent the clinical manifestation of a common disorder.

KEY MESSAGE: In summary, while morning or awakening headaches are reported to be common symptoms of OSA, studies failed to find a causal relationship between OSA and migraine. Nevertheless, evidence suggests that OSA may be a trigger for migraine in predisposed patients and may facilitate migraine progression. Nevertheless, further evidence is needed to support this hypothesis, to understand the possible underlying pathophysiological mechanism, and to clarify if the treatment of OSA patients with continuous positive airway pressure improves migraine through a better oxygenation supply, or indirectly via a higher quality of slow wave sleep and sleep efficiency.

**Fig. 3:**
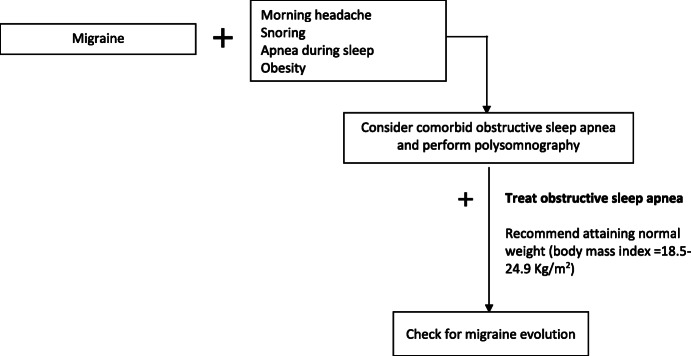
Interaction between migraine and obstructive sleep apnea. Although available studies failed to find a causal relationship between obstructive sleep apnea (OSA) and migraine, little evidence suggests that OSA may be a trigger of migraine in selected patients and facilitate migraine progression. For this reason it would be reasonable check for the presence of signs or symptoms attributable to OSA in migraine patients, especially in those reporting morning headache, habitual snoring, apnea episodes during sleep, obesity, craniofacial morphology and oral anatomy, neuromuscular disorders, and substances use. Clinicians should screen and select patients to be studied with polysomnography upon suspicion of sleep-related breathing disorder. Patients with OSA diagnosis should receive the guideline recommended treatments. Since available evidence suggests that obesity is a major risk factor for OSA development and progression and for migraine chronification, appraisal of a normal weight (body mass index=18.5-24.9 Kg/m^2^) should be strongly encouraged in patients with comorbid OSA and migraine since an improvement of both OSA severity and migraine frequency might be expected

The available evidence about migraine and OSA, although not supporting the existence of a clear association, suggests that it is reasonable to systematically check for the presence of signs or symptoms attributable to OSA in migraine patients and to treat OSA according to the current guidelines, since an improvement of migraine is also expected (Fig. 3). Patients reporting new-onset headache, or exacerbation of a preexisting primary headache, or morning headache, habitual snoring, witnessed apnea, and daytime sleepiness should be screened for sleep-related breathing disorders. Clinicians should be aware of the presence of known risk factors for sleep-related breathing disorders, including obesity, craniofacial morphology and oral anatomy, neuromuscular disorders and substances use. Targeted questions or specific questionnaires may help for screening patients and selecting those to be studied with PSG for the diagnosis of sleep-related breathing disorder. In presence of a sleep-related breathing disorder diagnosis, patients should receive the recommended treatments, including continuous positive airway pressure, surgery (eg uvulopalatopharyngoplasty or tonsillectomy), oral appliances, or conservative measures. Appraisal of a normal weight (body mass index = 18.5–24.9 Kg/m^2^) should be strongly encouraged in patients with comorbid OSA and migraine since an improvement of both OSA severity and migraine frequency might be expected.

## Sleep-related movement disorders

Sleep-related movement disorders are characterized by relatively simple, usually stereotyped, movements with consequent impairment of sleep or its onset [28, 29].

The most common sleep-related movement disorder is restless-leg syndrome (RLS), a condition characterized by an urge to move the legs, sometimes accompanied by an uncomfortable sensation that occurs primarily with rest or inactivity, partially or totally relieved by moving the legs for as long as the movement occurs, and occurring primarily in the evening or night, and closely associated with periodic limb movements (PLMs) [29]. General population studies found that the prevalence of RLS diagnosed according to the International Restless Legs Syndrome Study Group criteria [71, 72] ranged between 5.0% and 14.3% [73].

The comorbid association between migraine and RLS has been extensively investigated. Some studies evaluated the prevalence of migraine in patients with RLS [74–78], others the prevalence of RLS in patients with migraine [79–87].

The prevalence of migraine in subjects with RLS ranges from 12.6% to 53.2% [74, 75, 77, 78] and it is significantly higher compared to those without RLS [74, 75, 77]. Nevertheless, the results of a study [76] did not show a higher frequency of migraine-type headache in RLS patients when compared to its prevalence in the same population.

Results from a population-based-study performed on adults in a rural setting in Italy [77] showed that the association with RLS was specific for migraine, since the prevalence of migraine was higher in patients with RLS than in those without (12.6% vs 8.0%), while the prevalence of headache per se (54.4% vs 49.8%) or of tension-type headache (19.5% vs 23.0%) was similar in patients with and without RLS.

The prevalence of RLS in subjects with migraine ranges from 13.7% to 25% and it is significantly higher compared to those without [82–87] and to subjects with other primary headaches [79, 80].

The impact of RLS on migraine may be greater than the impact of migraine on RLS. Indeed, the presence of RLS was associated with a higher migraine headache severity [74], a higher occurrence of photophobia, phonophobia, nausea and dizziness [79], a higher migraine-related disability [74, 79, 82, 84], and higher scores to anxiety and depression scales [79, 80, 84, 86, 87] than those without RLS, while the presence of migraine was not associated with a significantly higher RLS severity [75, 86].

The association between RLS and migraine may be influenced by migraine attack frequency. A cross-sectional case-control study [81] observed a significant association between RLS prevalence and migraine frequency in patients with migraine with aura but not in those without aura. Moreover, a case-control study [82] found that RLS was more frequently reported by chronic than episodic migraineurs (34.3% vs 16%).

Evidence suggests that most antidepressant classes, including tricyclic, selective serotonin reuptake inhibitors, and serotonin-norepinephrine reuptake inhibitors are associated with the onset or worsening of RLS and PLMs [88–93]. Nevertheless, the question is still not fully answered since the evidence on this issue is limited and conflicting. For this reason, the American Academy of Sleep Medicine guidelines on the treatment of RLS and PLMs, published in 2012, made no specific recommendation about the avoidance of antidepressants [94]. The International Restless Legs Syndrome Study Group guidelines in 2016 recommended to assess the possible exacerbating effect of extrinsic factors, including antidepressants, on RLS symptoms [95].

The positive association between RLS and migraine has been hypothesized to be due to a shared dopaminergic dysfunction in the hypothalamic A11 nucleus (Fig. 1). Symptoms of migraine in the premonitory and headache phase, including yawning, drowsiness, mood changes, and irritability, the evidence of hypersensibility of migraineurs to dopaminergic agonists [96, 97], and the reversal effect of dopaminergic antagonists on the gastroparalysis induced by a migraine attack [98] suggest that dopamine may have a central role in the pathophysiology of migraine. The dopaminergic hypothesis of the comorbid association of migraine and RLS is further supported by data showing that migraineurs with RLS report premonitory symptoms significantly more often than migraineurs without RLS [99].

The A11 dopaminergic nucleus, through the D2-like receptors, modulate neuronal firing in the trigeminocervical complex, which is the major relay center for nociceptive afferent mediating migraine pain from the meninges and cervical structures to the hypothalamus that is linked with key areas of the pain neuroaxis, including the cortex, thalamus, and the brainstem [100]. Moreover, the A11 nucleus sends direct inhibitory projections to preganglionic sympathetic neurons, the dorsal horn and the motoneuronal site of the spinal cord [101, 102]. Therefore, dysfunction of the A11 dopaminergic nucleus facilitates the firing in the trigeminocervical complex and increases sympathetic activity in the spinal cord, possibly causing or worsening migraine and RLS.

**Fig. 4:**
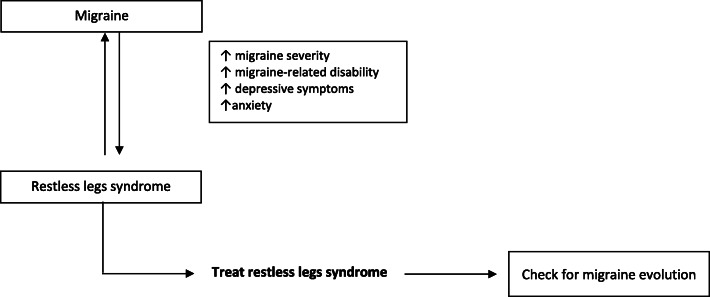
Interaction between migraine and restless legs syndrome. Available evidence suggests the existence of a bidirectional association between migraine and restless leg syndrome (RLS). RLS in patients with migraine seems to be associated with higher migraine frequency and related disability. It would be reasonable to systematically check patients with migraine for symptoms of RLS and adopt specific RLS treatments if needed; this approach should be considered complementary to that of migraine and may lead an improvement of migraine frequency and related disability. In the decision-making process for the choice of migraine preventive treatment clinicians should consider the possible exacerbating effect of antidepressants on RLS symptoms, and their effectiveness should be balance over the possible worsening effect on RLS

KEY MESSAGE: The available evidence suggests the existence of a strong bidirectional relationship between migraine and RLS (Fig. 4). Nevertheless, the available findings are too heterogeneous to support the existence of differences in the risk conferred by migraine with and without aura in the development of RLS. RLS in patients with migraine seems to be associated with higher migraine frequency and related disability. It would be reasonable to systematically check patients with migraine for symptoms of RLS and adopt specific RLS treatments if needed; this approach should be considered complementary to that of migraine and may lead an improvement in migraine frequency and related disability. While choosing the right migraine preventive treatment, clinicians should consider the possible exacerbating effect of antidepressants on RLS symptoms, and their effectiveness should be balanced over the possible worsening effect on RLS.

## Central disorders of hypersomnolence

Central disorders of hypersomnolence are characterized by excessive daytime sleepiness not caused by disturbed nocturnal sleep or by abnormalities of circadian rhythm. The cardinal feature of all the central disorders of hypersomnolence is a subjective complaint of excessive daytime sleepiness, defined as daily episodes of an irrepressible need for sleep or daytime lapses into sleep [29].

Narcolepsy has an estimated prevalence of 0.025% to 0.05% [103–105] with an age of onset varying from early childhood to the 50s with the first peak at 15 years and the second at 35 years [106].

After the early evidence of a high prevalence of migraine among narcoleptic patients reported by an observational study performed in 1999 [107], some other studies further deepened the relationship between these two pathologies. The same Authors, in a confirmatory study performed in 2003 [108], reported that the prevalence of migraine was 37% among patients with narcolepsy, observing the onset of narcolepsy symptoms to occur 12 years before the onset of migraine symptoms. Despite the high prevalence of migraine among narcoleptic patients, available evidence is conflicting and does not allow to establish the presence and the direction of this association. Indeed, a multicenter cross-sectional survey [109] found that migraine prevalence was significantly increased among the narcolepsy (23.5%) and idiopathic hypersomnia patients (41.2%) compared to the healthy control subjects (4.9%), while the prevalence of tension-type headache was similar among the narcoleptic, idiopathic hypersomnia and healthy control subjects. Contrarywise, a multicenter study [110] found an increased frequency of tension-type headache (60.3% vs. 40.7%) but not migraine (21.9% vs. 19.8%) in narcoleptic patients compared with controls.

A dysregulation of brainstem areas, including periaqueductal gray, the dorsal raphe nucleus, and the locus coeruleus has been proposed as a possible pathophysiological link between migraine and narcolepsy. Those regions participate to the transmission and modulation of pain in migraine [111–114], promote wakefulness and regulate sleep-wake sleep transition [115] (Fig. 1).

More recent data strongly suggests that the orexinergic system plays a crucial role in this association. Orexins are two neuropeptides, orexin A and B, synthesized in the lateral hypothalamus and implicated in the modulation of homeostatic functions, including appetite, sleep-wake, hormone secretion, and autonomic regulation [116–119]. The orexin peptides bind to two different receptors, OX1R and OX2R, and orexin levels are higher during wake periods. They actively enhance hypothalamic and brainstem neural networks to stimulate wakefulness [120], modulate the sleep-wake [121] and REM-non-rapid eye movement (NREM) sleep transition.

Dysfunction in the hypothalamic orexinergic system and loss of orexinergic neurons are well recognized pathological findings in subjects with narcolepsy, particularly in those with the form of narcolepsy with cataplexy, currently classified as narcolepsy type 1 [122]. Orexins, since they were shown to modulate the trigeminovascular tone, have received considerable attention also in migraine pathophysiology. Preclinical evidence found that orexin A have antinociceptive action, while orexin B have pro-nociceptive action and it seems to be driven by different pathway activated by the binding of orexins to OX1R and OX2R [123].

Overall, disrupted orexinergic systems can alter the homeostatic mechanism involved in nociception and sleep-wake control. Dual orexin receptor antagonists have been developed, and suvorexant has been approved for the treatment of insomnia and showed promising effective results for migraine treatment [124]. One randomized, double-blind, placebo-controlled clinical study has tested the efficacy of filorexant, another dual orexin receptor antagonist, for migraine prevention, but no statistically differences between treatment and placebo was found [125]. Further studies are required to clarify the possible efficacy of this drug class in migraine prevention and treatment of sleep disorders.

KEY MESSAGE: Migraine and narcolepsy are frequently comorbid but the available evidence does not allow full understanding of the strength and of the direction of this association. The pathophysiological mechanisms of both disorders seem to converge and involve the orexinergic system. The evidence of converging pathophysiological mechanisms may support the development of targeted treatment acting simultaneously on both conditions.

## Circadian rhythm sleep-wake disorders

Circadian rhythm sleep-wake disorders are defined as a chronic or recurrent condition of sleep-wake rhythm disturbance that may result from disruption of the endogenous circadian timing system or a misalignment between the endogenous circadian rhythm and the sleep-wake schedule desired or required by the social and physical environments [28, 29].

A Norwegian cross-sectional study on a nursing staff with different work schedules found that the prevalence of frequent headache (defined as ≥1 headache days per month), migraine, and chronic headache were higher among nurses with shift work disorder compared with nurses without shift work disorder [126]. Moreover, frequent headache, migraine, and chronic headache were significantly associated with shift work disorder (OR, 2.04; 1.60; 2.45 respectively) [126].

KEY MESSAGE: The limited number of available studies and the heterogeneity of the adopted methodology for the definition of circadian rhythm sleep-wake disorders do not allow reaching definitive conclusions about the relationship of these disorders with migraine. Further studies adopting more homogeneous diagnostic criteria should be conducted in order to investigate the possible role of circadian rhythm sleep-wake disorders on migraine onset and progression, and the effect of changes in scheduled timing required by social and physical environments on migraine burden.

## Parasomnias

Parasomnias are a group of sleep disorder characterized by undesirable complex movements, perceptions, behaviors, emotions, dreams and autonomic nervous system activity that may occur during entry into sleep, within sleep, or during arousal from sleep, and may disrupt the quality of sleep of the patient, the bed partner, or both [28, 29].

Suzuki et al. studied the association of migraine and REM sleep behavior disorder. In their case-control study authors demonstrated dream-enacting behaviors in 24.2% of patients with migraine compared to 14.3% in the control group [127]. The presence of dream-enacting behaviors was associated with higher headache-related disability and with lower sleep quality.

Data analysis from a large family study of the familial aggregation of anxiety and substance use disorders aimed at investigating the role comorbid anxiety and depression in the association between sleep problems and migraine [48], showed that adults with migraine had significantly more lifetime sleep problems and more current sleep difficulties, including persistent nightmares of childhood onset than those without migraine, and that this association was independent from both lifetime and current anxiety and mood disorders. At variance, sleepwalking and sleep enuresis were not associated with migraine.

Nevertheless, a cross-sectional case-control study [128] found that adult patients with PSG confirmed diagnosis of somnambulism (also known as sleepwalking) have a significantly increased risk of migraine and any headache type, and this relationship is independent from depression, daytime sleepiness, and insomnia severity. On the other hand, another cross-sectional case-control study found that, among adults with headache, a higher proportion of patients with migraine compared to those with other headache types (33% vs 5%) reported a childhood history of somnambulism [129].

A dysfunction of the serotoninergic pathway has been hypothesized as a possible common pathological mechanism because of the well-known role of serotonin in both sleep-wake regulation and in migraine pathogenesis. The most recent clinical hypothesis regards a dysfunction of orexinergic projections on the raphe nuclei that interfers with the serotonergic regulation, altering the nociceptive and the sleep regulating systems [130].

KEY MESSAGE: Available evidence seems to agree about the association between migraine and sleepwalking. Nevertheless, it is not possible to assess the strength and the type of association between migraine and parasomnias in adults because of the availability of only sparse studies.

## Conclusions

The relevance of the association between migraine and sleep disorders is underlined by evidence from epidemiological studies, by the intimate relationship in the clinical presentation and by the presence of shared anatomical pathways. Nevertheless, this relationship is confirmed to be complex and, although in recent years many studies improved our knowledge about it, there are still gaps that need to be bridged. Studies investigating anatomical structures and neuropeptides are crucial because provide insights into the underlying mechanisms involved in the relationship between migraine and sleep disorders, but might be also important for improving our knowledge about migraine pathology and for the development of new therapeutic approaches.

Although the strength of the relationship with migraine seems to differ according to the considered sleep disorder, the reported high prevalence of their comorbid association and their mutual exacerbation are important elements that should induce to implement the routine collection of sleep history and the administration of sleep quality questionnaires in headache centers. Diagnosis and treatment of comorbid sleep disorders should be considered in the management of migraine patients, since an improvement of sleep is expected to determine also a reduction of headache frequency and severity.

## Data Availability

All included references in the present review article are available on the Internet.

## Abbreviations

PRISMA: Preferred Reporting Items for Systematic Reviews and Meta-Analyses
ICDS: International Classification of Sleep Disorders
REM: rapid eye movement
NREM: non-rapid eye movement
PSG: Polysomnography
OR: odds ratio
RR: relative risk
RLS: restless-leg syndrome
PLMs: periodic limb movements
OSA: obstructive sleep apnea
